# Raman amplification at 2.2 μm in silicon core fibers with prospects for extended mid-infrared source generation

**DOI:** 10.1038/s41377-023-01250-y

**Published:** 2023-08-30

**Authors:** Meng Huang, Shiyu Sun, Than S. Saini, Qiang Fu, Lin Xu, Dong Wu, Haonan Ren, Li Shen, Thomas W. Hawkins, John Ballato, Anna C. Peacock

**Affiliations:** 1 Optoelectronics Research Centre, University of Southampton, Southampton, SO17 1BJ UK; 2 School of Optoelectronic Engineering and Instrumentation Science, Dalian University of Technology, Dalian, 116024 China; 3 Wuhan National Laboratory for Optoelectronics, Huazhong University of Science and Technology, Wuhan, 430074 China; 4 Center for Optical Materials Science and Engineering Technologies and Department of Materials Science and Engineering, Clemson University, Clemson, SC 29634 USA

**Keywords:** Mid-infrared photonics, Nonlinear optics

## Abstract

Raman scattering provides a convenient mechanism to generate or amplify light at wavelengths where gain is not otherwise available. When combined with recent advancements in high-power fiber lasers that operate at wavelengths ~2 μm, great opportunities exist for Raman systems that extend operation further into the mid-infrared regime for applications such as gas sensing, spectroscopy, and biomedical analyses. Here, a thulium-doped fiber laser is used to demonstrate Raman emission and amplification from a highly nonlinear silicon core fiber (SCF) platform at wavelengths beyond 2 μm. The SCF has been tapered to obtain a micrometer-sized core diameter (~1.6 μm) over a length of 6 cm, with losses as low as 0.2 dB cm^−1^. A maximum on-off peak gain of 30.4 dB was obtained using 10 W of peak pump power at 1.99 μm, with simulations indicating that the gain could be increased to up to ~50 dB by extending the SCF length. Simulations also show that by exploiting the large Raman gain and extended mid-infrared transparency of the SCF, cascaded Raman processes could yield tunable systems with practical output powers across the 2–5 μm range.

## Abstract (web summary)

This work demonstrates Raman amplification at 2.2 μm and the extension for mid-infrared source generation via cascaded processes by making use of a highly nonlinear silicon core fiber platform.

## Graphical abstract

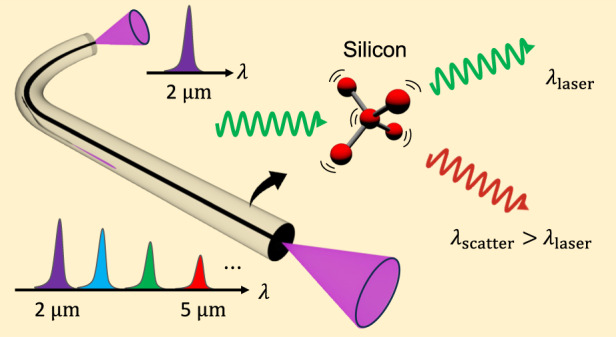

## Introduction

Compact and tunable light sources that can operate across the 2–5 μm regime are of great interest for gas sensing^1^, environmental monitoring^2^, and medical diagnostics^3^. To this end, fibers that are doped with rare-earth ions, such as thulium and holmium, have emerged as contenders for efficient light generation in this region^4^. As well as being robust and stable, these doped fiber systems offer key operational benefits such as large tensile strengths, flexible power scaling, and high-quality beam profiles with minimal thermal distortion. However, despite their great performance for wavelength emission near 2 μm, achieving high-power operation beyond 2.2 μm is challenging due to the need to switch from silicate to fluoride host glasses, owing to the lower phonon energies and higher transmission at longer wavelengths of the latter^5,6^. An alternative solution to extending the wavelength coverage of these sources is to make use of high-power emission at shorter wavelengths and shift the output via Raman scattering^7^. Importantly, compared to other nonlinear wavelength conversion processes such as four-wave mixing (FWM), Raman scattering is not restricted by phase-matching considerations^8^, so the newly generated wavelengths are only determined by the pump wavelength and the Stokes shift of the material. Thus, Raman amplifiers can be tuned to operate over a very broad wavelength range, with a gain bandwidth that can also be controlled by the bandwidth of the pump source^9^.

Compared to glass-based fibers, crystalline silicon waveguides are promising platforms for Raman processes due to their high damage threshold, strong Raman emission, and extended infrared transmission (1–8 μm). Significantly, Raman scattering in the telecom band was one of the first nonlinear processes demonstrated in a silicon waveguide^10^, and was closely followed by examples of amplification^9,11–13^ and lasing^7,14–16^. However, despite this initial success, and Raman amplification being demonstrated in bulk silicon at 3.4 μm^17^, currently Raman amplification or wavelength shifting in silicon waveguides has been confined to wavelengths <2 μm, which is attributed to the relatively short device lengths and limited power handling of the on-chip components^7^. In contrast, silicon core fibers (SCFs) have emerged as an alternative platform for Raman amplification that can offer extended propagation lengths, low propagation losses, and efficient coupling to fiber laser systems^18^. The SCFs are produced by a conventional fiber drawing method, which ensures high yields and low device costs^19^. Moreover, as they are clad in silica, the SCFs are robust, stable, and compatible with standard fiber post-processing methods such as tapering^20^ and splicing^21^, which allows for further optimization of the waveguide properties as well as seamless interconnection with other glass fiber components, such as the pump laser.

In this paper, high levels of Raman amplification are demonstrated at wavelengths >2 μm by making use of the long waveguide lengths of the highly nonlinear SCF platform. The SCF was tapered to achieve a low loss (~0.2 dB cm^−1^) nonlinear interaction region that consists of a constant tapered waist with a diameter of ~1.6 μm over a length of ~6 cm. A thulium-doped fiber laser that delivers picosecond pulses with a peak power of several watts at a wavelength of ~1.99 μm was used as a pump to generate a source of Raman-shifted photons at ~2.22 μm. The on-off gain for stimulated Raman amplification was estimated to be 30.4 dB, according to the measured time-averaged gain of 3.7 dB. By exploiting the low linear and nonlinear losses of the SCFs when pumped within the range 2.0–2.2 μm, simulations show the possibility to extend the reach of the Raman shifting to wavelengths >5 μm via a cascaded process. Thus, this work provides a crucial step toward the development of compact and tunable silicon-based Raman amplifiers for applications across the 2–5 μm regime.

## Results

### Fiber design and characterization

**Fig. 1:**
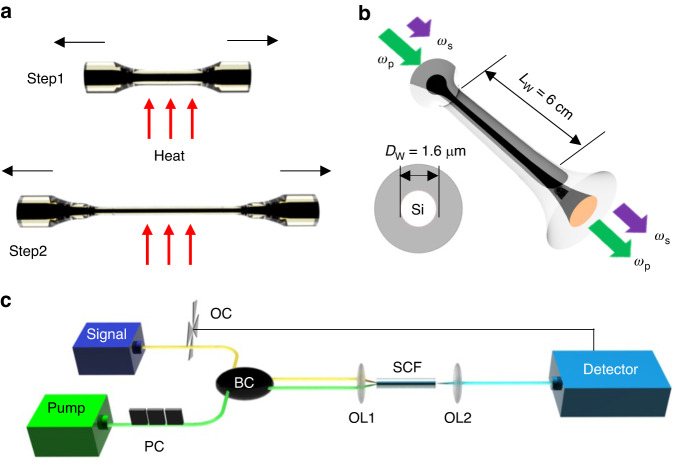
Fibre design, fabrication and measurement. **a** Schematic of the two-step tapering method for SCF optimization. **b** Schematic of tapered SCF as used for Raman scattering. **c** Experimental setup used for both spontaneous Raman scattering and stimulated Raman amplification measurements. OC optical chopper, BC beam combiner, PC polarization controller, OL optical lens, Detector optical spectrum analyzer/lock-in amplifier

The SCFs used in this work were fabricated via the molten core drawing method^19^, which produced fibers with uniform core/cladding diameters of 12 μm/125 μm and a polycrystalline core phase. To improve the nonlinear performance, the as-drawn fibers were subsequently tapered. As well as reducing the core size, the tapering also improves the crystallinity, which reduces the overall transmission losses (see Methods)^22^. Fig. 1a shows a schematic of the two-step tapering technique used to extend the tapered SCF waist length. By reducing the outer diameter in the first step, a lower filament power can be used in the second taper, which is important for producing micrometer-sized continuous cores with long single-crystal grains. Using this method, a SCF was fabricated with a waist length of 6 cm, which is the longest tapered SCF produced to date. A schematic of the final tapered SCF geometry is shown in Fig. 1b, in which the tapered waist region has a core diameter of 1.6 μm, and is positioned between two taper transition regions. The taper transitions are included to improve the SCF coupling, and scale up to input/output core diameters of ~4.6 μm over lengths of ~2.5 mm, resulting in a total SCF length of 6.5 cm. The target waist diameter for this work was slightly larger than that used in previous experiments demonstrating Raman amplification in the telecom band^18^, to ensure low transmission loss for the longer wavelength pump and Raman shifted signal. Before conducting the measurements, the input and output facets were polished using a routine fiber preparation method to ensure optimal coupling can be achieved.

The experimental setup is shown in Fig. 1c. A gain-switched laser diode (Eblana Photonics) seeded thulium-doped fiber master oscillator power amplifier (Tm: MOPA) system was used as a pump laser source^23^. The pump has a ~125 ps full width at half maximum (FWHM) pulse duration with a repetition rate of 10 MHz and delivered 70 mW of average power (maximum peak power of ~56 W) at 1.99 μm. The signal used for Raman amplification is a continuous wave (CW) mid-infrared laser (Cr^+2^: ZnS/Se IPG Photonics) tunable over the range of 2.0–2.4 μm, with a minimum wavelength resolution of 0.3 nm. To combine the pump and signal before injection into the tapered SCF, a 90:10 fiberized beam combiner (BC) was used. The combined lasers were launched into the fundamental mode of the SCF using a ×40 objective lens (OL1, NA: 0.65), with the aid of a camera to monitor the beam profile, and the output pump and Stokes wave were collected with a ×60 lens (OL2, NA: 0.85). As discussed in Supplementary Information Section I, the estimated transmission loss translates to a propagation loss (*α*) of only ~0.2 dB cm^−1^, which is comparable to the lowest losses obtained in the SCF platform^24,25^. To characterize the spontaneous Raman scattering, the signal laser was turned off and the output light was sent to an optical spectrum analyzer (OSA-Yokogawa AQ6375) via a mid-infrared patch cord (Thorlabs M42). For characterization of the Raman amplification, the CW signal was tuned across the measured spontaneous Stokes wave bandwidth. An optical chopper was used to modulate the DC signal before coupling into the fiberized BC, and a lock-in amplifier (LIA) was used to detect the power variation of the output signal. It is worth noting that for photon wavelengths with energies greater than half the bandgap of silicon (*E*_g_ = 1.12 eV), two-photon absorption (TPA) and TPA-induced free carrier absorption (FCA) play important roles in nonlinear silicon processes. The TPA coefficient (*β*_TPA_) at the pump wavelength of 1.99 μm has been previously characterized in the tapered SCFs to be ~0.3 cm GW^−1^, which is around half as strong as the value at 1.55 μm (~0.7 cm GW^−1^)^26^.

### Spontaneous Raman scattering

**Fig. 2:**
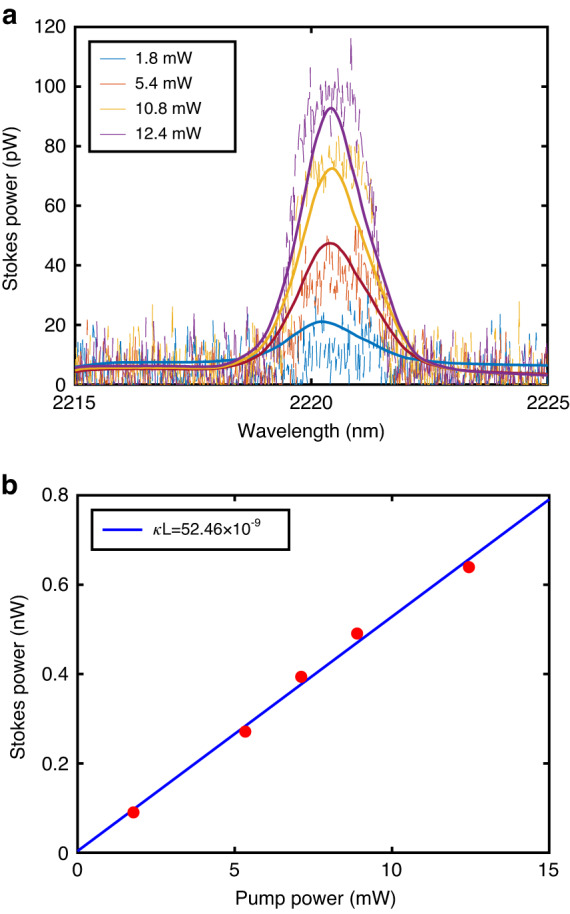
Spontaneous Raman scattering at 2.2 μm. **a** Spontaneous Raman emission spectra at various time-averaged pump powers, as given in the legends, for a pump wavelength of 1.99 μm. **b** Spontaneous Stokes power as a function of coupled-in average pump power

To characterize the spontaneous Raman scattering, the spectral output from the tapered SCF was monitored via the OSA as a function of input pump power with a sensitivity setting of HIGH2/CHOP mode. Fig. 2a shows the appearance of the spontaneous Stokes wave for average powers as low as ~2 mW. The Stokes peak is positioned at ~2.22 μm, corresponding to the expected Raman shift of 15.6 THz. Due to the narrow linewidth of the pump source (<0.12 nm), the linewidth of the Stokes wave (~1.7 nm) is close to the intrinsic bandwidth (105 GHz) of the Raman emission for the silicon core. The SCF parameters that correspond to the experiments are given in Supplementary Information Section II, together with the generalized nonlinear Schrödinger equation, including the Raman response function, used to model the measured data^26^. The simulation results are plotted together with the measurements in Fig. 2a, showing excellent agreement. To compare the efficiency of the spontaneous Raman emission with previous results obtained in the telecom band, the relationship between the integrated Stokes power versus pump power is considered^27^:

$${P}_{{\rm{s}}}=\kappa {L}_{{\rm{eff}}}{P}_{{\rm{p}}}$$

Here *κ* is the spontaneous Raman coefficient in units of cm^−1^ and *L*_eff_ is the effective length (${L}_{{\rm{eff}}}=(1-{e}^{-\alpha L})/\alpha$) of the tapered SCF. Fig. 2b plots the generated Stokes power as a function of coupled-in average pump power, from which *κ* can be determined as 8.7 × 10^−9^ cm^−1^ from the linear fit. The spontaneous Raman efficiency *S* in the SCF can then be estimated to be ~1.05 × 10^−7^ cm^−1^ Sr^−1^. Comparing this value with previous results for 1.43 μm pump sources, where *S* = 3 × 10^−7^ cm^−1^ Sr^−1^ was obtained for the SCF^18^ and *S* = 4.1 × 10^−7^ cm^−1^ Sr^−1^ in a typical planar waveguide^10^, the lower *S* is in agreement with the *λ*^−4^ dependence.

The spontaneous Raman efficiency can then be used to calculate the expected Raman gain coefficient (*g*_s_, in units of cm GW^−1^) at the pump wavelength via the equation^27^:

$${g}_{\rm{s}}=\frac{8\pi {c}^{2}{\omega }_{\rm{p}}}{{\hslash}{\omega }_{\rm{s}}^{4}{n}^{2}\left({\omega }_{\rm{s}}\right)\left(N+1\right)\Delta {\omega}}S$$

Here *ω*_p_ and *ω*_s_ are the angular frequencies of the pump and Stokes signals, respectively, *n* is the refractive index, *N* is the Bose occupation factor (0.1 at room temperature), ℏ is Planck’s constant divided by 2*π*, and Δ*ω* is the FWHM bandwidth of the Raman response in silicon. The value of *g*_s_ is found to be 18 cm GW^−1^ at the pump wavelength of 1.99 μm. Similar to *S*, *g*_s_ follows the expected wavelength trend, which is to decrease with a *λ*^−1^ dependency, so that the value here is lower than previous reports for the telecom band^10^ but higher than the value obtained in bulk silicon for a pump at 3.4 μm^17^. However, the slightly lower *g*_s_ is expected to be compensated by the lower nonlinear absorption for the 1.99 μm pump, so that higher pump peak powers can be used^28^.

### Stimulated Raman amplification

**Fig. 3:**
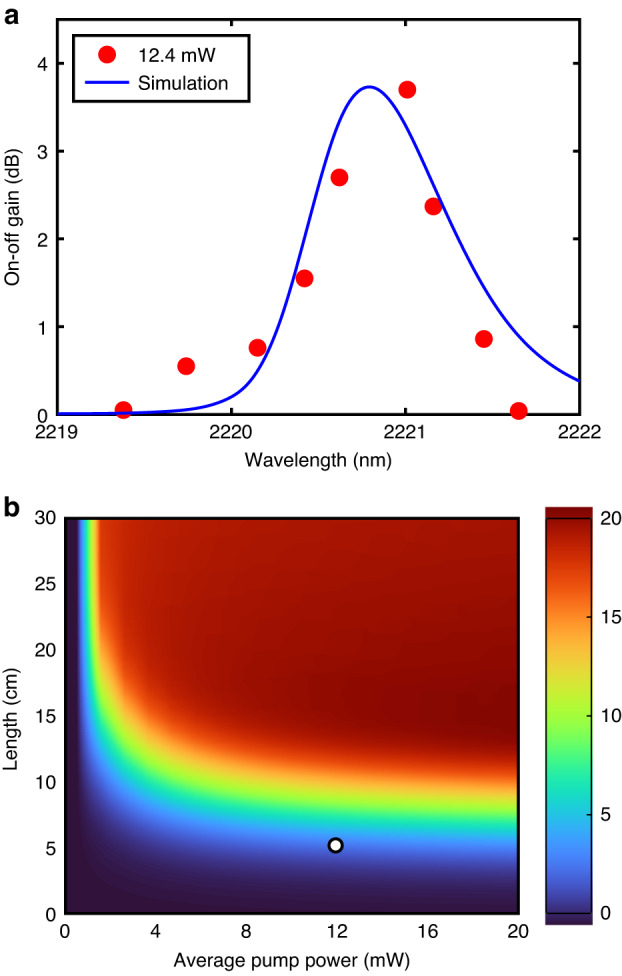
Stimulated Raman amplification at 2.2 μm. **a** Stimulated Raman gain for a 1.99 μm pulse pump with 12.4 mW of coupled power for various signal wavelengths. **b** Simulation results of on-off gain as a function of coupled pump power and waist length of SCF

With the estimated Raman gain coefficient, investigations subsequently turned to the observation of forward-stimulated Raman amplification. To demonstrate the capacity for efficient Raman amplification in this 2 μm wavelength region, the same experimental setup was employed, but with the coupled-in pump power fixed at 12.4 mW (corresponding to a peak power of 10 W), and an input signal power of 0.1 mW. Although the stimulated Raman gain was easily observable for average pump powers as low as ~3 mW, the highest pump power was selected to obtain the largest measurable gain, which is more important for future applications. The measured time-averaged on-off gain as the signal wavelength is tuned across the Raman gain curve is shown in Fig. 3a, together with simulation results that use the parameters obtained via the spontaneous measurement. A maximum time-averaged gain of 3.7 dB was measured for the signal wavelength of 2.22 μm, with a measured average signal power of ~10 nW out of this system. Owing to the pulsed nature of the pump beam, the amplified signal will also occur as a train of short pulses^29^. By converting the time-averaged on-off gain using the duty cycle factor (*F* = 1/(10 MHz∙125 ps)), the peak pulse gain is calculated to be ~30.4 dB, corresponding to a peak signal output of 0.3 mW. Significantly, this gain is substantially larger than the 12 dB that was reported for amplification at 3.4 μm in bulk silicon, which is attributed to the higher pump intensity used here (~900 MW cm^−2^ vs. 217 MW cm^−2^) and longer nonlinear interaction length (6.5 cm vs. 2.5 cm) available via the fiber platform^17^. Moreover, this gain is also significantly higher than that previously obtained in the telecom band for the SCFs platform (~1.1 dB for CW pumping^18^), and only slightly lower than the best results for conventional on-chip waveguides for a pump power more than five times larger (6.8 dB measured gain, corresponding 45.8 dB on-off gain for a 6.6 ps pump pulse with a peak-coupled power of 55 W^30^). We note that although the gain bandwidth of the stimulated amplification in this crystalline core fiber is much narrower than the bandwidths for glass fibers, this could be expanded by using a broadband pump source or even multiple pumps^9^, and the larger gain coefficient associated with the narrow Raman response is advantageous for improving the efficiency.

The noise figure (NF) of this SCF Raman amplifier was then estimated by comparing the input and output signal-to-noise ratio to be ~10 dB. Although this is slightly better than previous demonstrations of silicon Raman amplifiers in the telecom band due to the lower losses^31^, it is higher than what has been achieved in some of the highly nonlinear glass mid-infrared fiber amplifiers that use much longer waveguide lengths (up to a few km)^32^. However, similarly low NFs could be obtained using the SCFs with a slight increase in the length to ~10 cm, thus maintaining the compactness of the system. We note that although setups, where the pump is launched counter-propagating to the signal, are typically preferred in communication systems, both to avoid residual pump photons at the output and to lower the noise properties of the amplified signal, this introduces more complexity to the system. Moreover, as the Raman gain is similar for both forward and backward pumped amplification in our short SCF (~6 cm), a higher gain has in fact been obtained for forward pumping due to the lower losses in our co-propagating setup (see Supplementary information III).

To further probe the Raman amplification performance of the SCFs within the current system, additional simulations were conducted to investigate the role of the pump power and the fiber length. Fig. 3b plots the predicted time-averaged on–off gain, assuming that the remaining SCF and pulse parameters are the same as the experimental procedure herein. The maximum measured gain obtained in Fig. 3a is also labeled on the colormap for ease of comparison. Interestingly, due to the non-negligible TPA parameter at the 1.99 μm pump wavelength, these results show that there is little benefit in increasing the pump power much beyond the existing value due to the substantial FCA associated with the 125 ps pump pulse. In fact, to directly compare with the earlier short-pulsed telecom system of ref. ^30^, increasing the peak power in this system to 55 W would result in a time-averaged gain of only 6 dB. However, increasing the SCF length to 20 cm while retaining the same pump power, does result in a substantial increase in the time-averaged on-off gain up to ~20 dB (corresponding to a peak on–off gain of 49 dB), which would result in average signal powers as high as 1 μW (peak power of 0.8 mW). Thus, this analysis highlights the importance of optimizing the system to minimize the role of nonlinear absorption processes to obtain high gains, and thus high signal output powers, as will be discussed below.

### High power and tunable systems

To explore the potential to generate higher power and longer wavelength sources using this SCF Raman system, additional simulations were conducted to investigate the conditions for efficient cascaded Raman scattering. As described in Supplementary Information Section IV-a, the experimental pump wavelength is close to the zero-dispersion wavelength (ZDW). Therefore, the first step was to study the optimum core diameter of the SCF waist to shift the ZDW and ensure that Raman scattering was the dominant nonlinear conversion process. Although Raman processes do not require phase-matching, when operating close to the ZDW, competition from FWM can result in suppression of the Raman gain^33^. Significantly, increasing the core waist diameter slightly to 1.7 μm, which positions the pump further from the ZDW, can result in three orders of magnitude enhancement in conversion to the first and second-order Stokes waves (see Supplementary Information Section IV-b). The second step involved investigating the role of nonlinear absorption, and specifically the build-up of free carriers associated with the long pulse duration, which was shown to be a limiting factor to increasing the gain in Fig. 3b. By reducing the pulse duration to 40 ps, it is possible to reduce the FCA contribution to increase the generated Stokes power for a similar level of average input power (see Supplementary Information Section V).

**Fig. 4:**
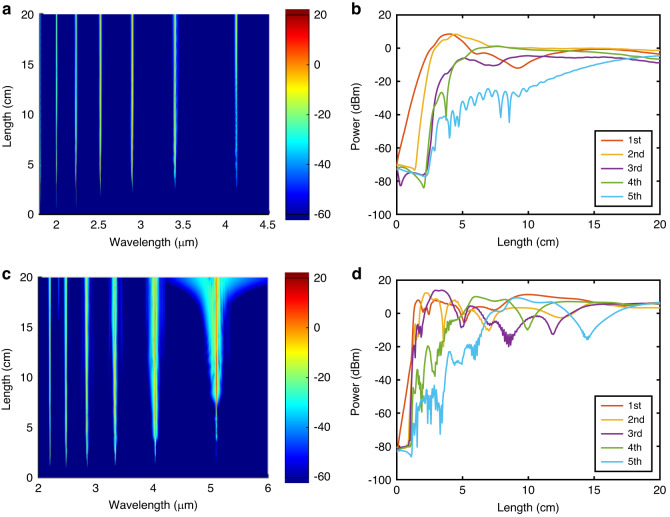
Cascaded Raman scattering for wavelength conversion across 2-5 μm. **a** Simulated spectral evolution of cascaded Raman scattering with 2 μm pulsed laser pump. **b** Output peak powers of Stokes waves as a function of fiber length for Fig. 4a. **c** Simulated spectral evolution with 2.2 μm pulsed laser pump. **d** Output Stokes peak powers as a function of fiber length for Fig. 4c

Using these new values of the waist diameter and pulse duration, Fig. 4a shows the spontaneous Stokes power generated as a function of wavelength and fiber length assuming a coupled-in average pump power of 8 mW (peak power of 20 W). Although the average power is slightly lower than the maximum value used in the experiments, the peak power is twice as high owing to the shorter pulse duration. Fig. 4b shows the output spontaneous Stokes powers as a function of fiber length. As can be seen in Fig. 4b, at a propagation distance of 2.5 cm, the first-order Stokes wave has intensified to have a similar power level with the pump, which enables it to act as a pump for the second-order Stokes wave at 2.5 μm. The second-order Stokes wave then grows to have a maximum power at the propagation distance of 4.5 cm and acts as a pump for the third-order Stokes wave. Moreover, as the fiber length increases further, Raman Stokes waves out to the 5th order can be generated (λ ~ 4.1 μm) with a high peak power level (>0.3 mW). The maximum output peak powers for the Stokes wave from 1st order to 5th order are 7.10 mW, 6.90 mW, 0.35 mW, 1.27 mW, and 0.34 mW, corresponding to output average powers of 3.18 μW, 2.76 μW, 0.14 μW, 0.51 μW, and 0.14 μW, respectively.

To increase the powers and extend the wavelength coverage further, it is also worth exploring the benefits of switching to a slightly longer pump wavelength, such as could be offered by a holmium-doped fiber system operating at 2.2 μm^34^. Interestingly, 2.2 μm represents a favorable wavelength for pumping nonlinear processes in silicon as it is at the edge of the TPA region, so that *β*_TPA_ is negligible. This wavelength also is shorter than where higher-order three-photon absorption processes become significant. Thus, one can expect the nonlinear absorption to play a minimal role for such pump sources and the original pump pulse duration of 125 ps can be used to increase the energy in the generated Stokes waves. Fig. 4c plots the spontaneous Stokes power as a function of wavelength for a 2.2 μm pump, assuming the same fiber length and coupled input peak power (20 W) as in Fig. 4a, clearly showing the strongly cascaded conversion. The output peak powers of the generated Stokes waves as a function of fiber length are then plotted Fig. 4d. Due to the significant reduction in TPA and FCA for this wavelength, a substantial increase in the conversion efficiency is observed, with the 2nd-order Stokes wave (now at a wavelength of ~2.8 μm) appearing after only 2 cm of propagation. Moreover, as the 2nd-order Stokes power increases, the process can continue to rapidly generate higher-order Stokes waves (up to the 5th order at *λ* ~5.1 μm) for fiber lengths of only ~5 cm. Moreover, the Raman wavelength conversions essentially all happen within the first 10 cm, so there is little no benefit to extending the SCF length for 2.2 μm system beyond this. Thanks to the negligible nonlinear absorption and increased pump pulse energy for the 2.2 μm system, the obtainable maximum peak powers of all Raman Stokes waves now exceed 8.67 mW, corresponding to an average power of 10.80 μW, which is two orders of magnitude higher than 2 μm system. Further, these powers could be increased by an order of magnitude with increasing input pump powers^35^. Thus, these results show the potential to extend the wavelength coverage of the SCF-based Raman system across the 2–5 μm wavelength region, and beyond^36^.

## Discussion

In summary, this work reports the generation of spontaneous Raman scattering and stimulated amplification extending beyond 2 μm using a highly nonlinear SCF. The fiber has been tapered to achieve a micrometer-sized core with low propagation losses of 0.2 dB cm^−1^ at the 1.99 μm pump wavelength, over an extended length of 6 cm. The combination of relatively high Raman gains and low nonlinear absorption around the 2.0–2.2 μm wavelength region, where many high-power pump lasers exist, allows for efficient wavelength conversion and amplification, with ~30.4 dB of on-off gain using ~10 W of peak pump power. Moreover, simulations of the nonlinear propagation suggest that the performance can be optimized by altering the SCF core diameter for the specific pump wavelength to ensure that Raman scattering is the dominant nonlinear process, as well as minimizing the impact of FCA. Specifically, shown here is the possibility to extend the generated signals to wavelengths of ~4 μm for a 2 μm pump source by accessing cascaded Raman processes, and even further to ~5 μm when using a source of wavelength longer than 2.2 μm. The ability to tune the signals across the 2–5 μm wavelength region confirms the highly nonlinear SCF platform is a suitable candidate for all-fiber integrated Raman amplifiers or lasers in the mid-infrared regime, and thus would be a useful source for applications in gas sensing^1–3^, free-space communications^37^ and biological imaging systems^38^. Moreover, compared to Raman amplifiers developed from highly nonlinear glass fibers such as the chalcogenide materials^39^, the high damage thresholds and large Raman coefficient (>10 times larger) associated with the crystalline material allows for the construction of robust and compact high-power systems that are still compatible with all-fiber infrastructures.

## Methods

### Two-step tapering

The tapering process in this work was realized by using a standard glass processing system (Vytran GPX3300), during which the fiber is heated and stretched. As the silicon core is completely molten during this process, tapering provides a means to increase the crystal grain length from millimeters to centimeters by controlling the cooling dynamics of the core during the recrystallization process^22^. The improved crystallinity results in a reduction of the transmission losses by around an order of magnitude. However, as the final core diameter of the tapered SCF used in this work (1.6 μm) is much smaller than that in the as-drawn fibers (12 μm), a two-step tapering method was used for the production process. In the first step, a tapering ratio of 125/50 was used to produce SCFs with a core/cladding diameter of 4.8 μm/50 μm. In the second step, the tapering process starts within the uniform waist region produced by the first step and the tapering ratio is set to 80/25. The drawing speeds are 1 mm s^−1^ for both steps but the filament power of the second step (~55 W) can be set 10 W lower than the first step (~65 W). Compared to the single-step method, two-step tapering can make use of a smaller tapering ratio and lower thermal energy in the final process, which is important for producing continuous lengths of high-quality single-crystal silicon cores. It is worth noting that due to the large core/cladding index difference, the SCFs can sustain larger taper ratios (tan *θ* ≤ 0.2) than traditional all-silica fibers. Therefore, the down and up tapering regions are designed to have lengths of 3 mm to achieve low loss and retain single-mode propagation (see Supplementary Information Section I)^40^. After tapering, the processed SCF is mounted inside a thick polymer capillary, which has an inner diameter of 250 μm and outer diameter of 665 μm by using wax (crystal bond 509). Then a standard polishing process is applied to polish the fiber facet for efficient light coupling.

### Supplementary information

supplementary for the paper

## Data Availability

10.5258/SOTON/D2549
