# Supplementary material for: Raman amplification at 2.2 μm in silicon core fibers with prospects for extended mid-infrared source generation
Source: Light Sci Appl. 2023 Aug 30;12:209. doi: 10.1038/s41377-023-01250-y (PMC10469167; doi:10.1038/s41377-023-01250-y)
Supplement: Supplementary file 1 — supplementary for the paper [file 41377_2023_1250_MOESM1_ESM.docx]

**Supplementary Information:**

**Raman Amplification at 2.2 μm in Silicon Core Fibers with Prospects for Extended Mid-infrared Source Generation**

Meng Huang^1,*^, Shiyu Sun^1,*^, Than S. Saini^1^, Qiang Fu^1^, Lin Xu^1^, Dong Wu^1^, Haonan Ren^2^, Li Shen^3^, Thomas W. Hawkins^4^, John Ballato^4^ and Anna C. Peacock^1^

^1^Optoelectronics Research Centre, University of Southampton, Southampton, SO17 1BJ, United Kingdom

^2^School of Optoelectronic Engineering and Instrumentation Science, Dalian University of Technology, Dalian, 116024, China

^3^Wuhan National Laboratory for Optoelectronics, Huazhong University of Science and Technology, Wuhan, 430074, China

^4^Center for Optical Materials Science and Engineering Technologies and Department of Materials Science and Engineering, Clemson University, Clemson, South Carolina 29634, USA

**Corresponding author:*

*Meng Huang:* [*m.huang@soton.ac.uk*](mailto:m.huang@soton.ac.uk)*; (+44)7529932813*

*Shiyu Sun:* [*s.sun@soton.ac.uk*](mailto:s.sun@soton.ac.uk)*; (+44)7598414168*

1. **Propagation loss estimation**

To characterise the propagation loss of the tapered silicon core fiber (SCF), the insertion loss of the device was measured with the picosecond laser at 1.99 $\mu m$, using the lowest power setting to minimise the effect of nonlinear absorption. The combined lasers were launched into the SCF using a 40X objective lens (OL1, NA: 0.65), and the output pump and Stokes wave were collected with a 60X lens (OL2, NA: 0.85). Cameras were used at the input and output coupling lenses to monitor the mode profile to ensure that the light is coupled predominantly into the fundamental mode^1^. The measured insertion loss for this SCF was 12.8 dB and the loss contributions are summarized in Table S1.

**Table S1** Summary of loss contribution in the insertion loss measurement

| Contributor | Details | Loss (dB) |
| --- | --- | --- |
| OL1 | M40 Newport (NA 0.65, lens diameter 5 mm) | 2 |
| OL2 | M60 Newport (NA 0.85, lens diameter 5 mm) | 1.5 |
| Coupled-in loss | OL1 to input facet | 3.5 |
| Coupled-out loss | Output facet to OL2 | 1.5 |
| Facet reflection | Air to silicon or silicon to air | 1.5 |
| Fiber propagation | 0.2 dB cm^-1^ over a length of 6.5 cm | 1.3 |

Here the coupled-in and out loss are estimated through calculating the overlap between the focused beam size of lenses and the profile of fundamental mode in SCF using Mode Solution.

1. **Fiber parameters and generalized nonlinear Schrödinger equation (GNLSE)**

**Table S2** Parameters for spontaneous simulation

| Parameter | Label | Value | Parameter | Label | Value |
| --- | --- | --- | --- | --- | --- |
| Core size | $D_{w}$ | 1.6 $\mu m$ | Input loss | $\alpha_{\mathrm{in}}$ | 7.5 dB |
| Linear loss | $\alpha$ | 0.2 dB cm^-1^ | Output loss | $\alpha_{\mathrm{out}}$ | 4 dB |
| GVD | $\beta_{2}$ | 0.003 ps^2^ m^-1^ | Nonlinear index | $n_{2}$ | 6 $\times$ 10^-18^ m^2^ W^-1^ |
| FOD | $\beta_{4}$ | -7.23 $\times$ 10^-7^ ps^4^ m^-1^ | Free carrier dispersion | $\mu$ | 5.87 |
| Effective mode area | $A_{\mathrm{eff}}$ | 1.17 ${\mu m}^{2}$ | phonon lifetime | $\tau_{1}$ | 7.5 ns |
| TPA coefficient | $\beta_{\mathrm{TPA}}$ | 0.3 cm GW^-1^ | damping time | $\tau_{2}$ | $3.5$ ps |
| Free carrier loss | $\sigma$ | 1.45 $\times$ 10^-21^ m^2^ | Response function | $f_{R}$ | 0.038 |

Simulations were conducted by using the generalized nonlinear Schrödinger equation (GNLSE)^2^:

$\frac{\partial A}{\partial z}+\frac{\alpha}{2}A-i\sum_{n=1}^{\infty} \frac{i^{n}\beta_{n}}{n!}\frac{\partial^{n}A}{\partial t^{n}}-\frac{\sigma}{2}\left( 1+i\mu\right)N_{c}A=i(\gamma\left( \omega_{0} \right)+i\gamma_{1}\frac{\partial}{\partial t})(A(z,t)\int_{0}^{\infty} R(t^{'})\left| A(z,t-t^{'}) \right|^{2}dt^{'})$

Here $A$ is the pulse amplitude; $\alpha$ is the linear propagation loss; $\beta_{n}$ is the *n*^th^ order dispersive term; $\gamma={\omega_{0}n_{2}}/{cA_{\mathrm{eff}}}+i{\beta_{\mathrm{TPA}}}/{2A_{\mathrm{eff}}}$ is the nonlinearity, $n_{2}$ is the nonlinear refractive index, $A_{\mathrm{eff}}$ is the effective mode area;$\gamma_{1}={(d\gamma/d\omega)}_{\omega=\omega_{0}}$; $\sigma$ is the free carrier loss , $\mu$ is the free carrier dispersion. $N_{c}$ is the free carrier density which is governed by the following equation:

$\frac{\partial N_{c}(t)}{\partial t}=\frac{\beta_{TPA}}{2\hbar\omega_{0}}\frac{\left| E(t) \right|^{4}}{A_{eff}^{2}}-\frac{N_{c}(t)}{\tau}$

Here $\beta_{\mathrm{TPA}}$ is the two-photon absorption coefficient and $\tau$ is the free-carrier lifetime. The nonlinear response function $R\left( t \right)$ is given by:

$R\left( t \right)=\left( 1-f_{R} \right)\delta\left( t \right)+f_{R}h_{R}(t)$

where $f_{R}=g_{R}\Gamma_{R}/(n_{2}k_{0}\Omega_{R})$ represents the fractional contribution of the delayed Raman response to the nonlinear polarization in which $g_{R}$ is the Raman gain, $\Gamma_{R}/\pi$ is the gain bandwidth, $\Omega_{R}/2\pi$ is the peak gain frequency shift. Raman response function is given by: $h_{R}\left( t \right)=(\tau_{1}^{-2}+\tau_{2}^{-2})\tau_{1}exp(-t/\tau_{2})sin(t/\tau_{1})$, in which $\tau_{1}$ and $\tau_{2}$ is the Raman damping time of Raman vibrations.

1. **Backward spontaneous Raman emission**

Backward spontaneous Raman emission was measured by introducing a fiberized beam splitter before the SCF. The results are shown in Fig. S1. Due to the higher system losses in this configuration where a fiberized beam splitter (75: 25) is used to couple pump (75%) into the SCF with a tapered lens fiber and couple out the back Raman scattering signal with the other port (25%), the measured backward Raman power is lower than forward scattering (around 4 dB lower). Although the backward Raman amplification is often favored in traditional systems as it can avoid residual pump power at the output and it also lowers the noise properties, owing to the short length of the SCF used in our experiments ($\sim$6 cm), the Raman gain and noise properties for our system are in fact similar for the forwards and backward pumped configurations. Therefore, only forward direction pumping is investigated for Raman amplification.

**Figure S1** Recorded backward spontaneous Raman emission spectrums at various time-averaged pump powers, as given in the legends, for a pump wavelength of 1.99 μm.

1. **The influence of fiber dispersion for Raman efficiency**
2. Figure S2 plots the simulated group velocity dispersion (GVD) profiles in the SCFs. For the fiber core size (*D* = 1.6 $\mu$m) we used in experiment, the pump wavelength is very close to the zero-dispersion wavelength (ZDW), where the four-wave mixing (FWM) process could be strong^3^. However, as shown in Fig. S2, the ZDW can be shifted by varying the core diameter.

**Figure S2** Calculated group velocity dispersion (GVD) for the SCF with different core diameters shown in the legend.

1. By using the parameters from Table S2 and the calculated values of the dispersion, Fig. S3 shows the output peak power of Stokes waves as a function of fiber core diameter. The Stokes waves are generated via spontaneous cascaded Raman scattering, assuming the peak power of the pump is 10 W, and the pulse duration is 125 ps, as used in the experiments. The cascaded Raman scattering reaches a minimum for a core diameter of 1.58 $\mu m$. This is attributed to the strong competition with the FWM process, which overlaps with the Raman bandwidth^2^. However, when increasing the core diameter to 1.7 $\mu m$, even the 5^th^ order Stokes wave (4.1 $\mu m$) can achieve maximum output power. Although increasing the core diameter further, allows for the observation of the 6^th^ order Stokes wave (>5.5 $\mu m$), the converted powers for all the other waves are significantly reduced. Therefore, 1.7 $\mu m$ was chosen for long wavelength generation through cascaded Raman processes in the additional simulations.

**Figure S3** Cascaded Raman scattering output Stokes power as a function of fiber core diameter, for the Stokes order given in the legend.

1. **Raman efficiency simulation as a function of pulse width**

When pumping at 2 $\mu m$ with a fixed peak power of 10 W, Fig. S4 shows the output Stokes peak powers from cascaded Raman scattering as a function of pump pulse width for a 1.7 $\mu m$ core diameter fiber. As we can see, the high-order Stokes waves (2^nd^, 3^rd^, 4^th^, 5^th^) achieve a maximum output power when decreasing the pulse width to 40 ps. Therefore, we choose a pulse width of 40 ps (FWHM) width for simulations of cascaded Raman scattering for the 2 $\mu m$ pump.

**Figure S4** Cascaded Raman scattering output Stokes powers as a function of pulse width, for the Stokes order given in the legend.

**Supplementary reference**

1 Peacock, A. C. *et al.* Nonlinear pulse dynamics in multimode silicon core optical fibers. *Opt. Lett.* **37**, 3351-3353 (2012).

2 Agrawal, G. P. Nonlinear fiber optics. in *Nonlinear Science at the Dawn of the 21st Century* 195-211 (Springer, 2000).

3 Lin, Q. *et al.* Ultrabroadband parametric generation and wavelength conversion in silicon waveguides. *Opt. Express* **14**, 4786-4799 (2006).
